# Fellow-Workers and Their Doings

**Published:** 1914-07-11

**Authors:** 


### ROUND THE WORLD.

### Fellow-Workers and their Doings.

[The Editor Invites Early Information and Contributions Marked "Fellow-Workers."]

After waiting many years for a staff appointment at a teaching hospital it has been the good fortune of Dr. Thomas G. Stevens to cover his out-patient work and find himself promoted to the position of senior obstetric physician within the short space of two years. Dr. Stevens did a good deal of teaching as a junior at Guy's before going to St. Mary's as obstetric tutor, and then had to wait about ten years before he became a member of the staff at the latter institution on the retirement of Dr. Handfield Jones, whose successor has himself now retired. Dr. T. G. Stevens is an M.D. Lond., and also holds the Fellowship of both Royal Colleges; he is surgeon to the Hospital for Women, Soho, and in-patient physician to Queen Charlotte's Hospital.

Dr. William John Gow, whose resignation from the staff of St. Mary's Hospital has opened the way for Dr. T. G. Stevens, is generally recognised as a brilliant exponent of obstetric surgery. As a student at "Bart.'s" he won many distinctions, including the gold medal in medicine and first-class honours in obstetrics at the London M.D. examination; subsequently he attached himself to St. Mary's Hospital, where he has been well known as a successful operator; he is also consulting physician to Queen Charlotte's Hospital and consulting gynæcologist to the Royal Waterloo Hospital.

Sir Alexander Ogston, K.C.V.O., M.D., who has been invited by his old dressers and surgeons to a luncheon to be held at Aberdeen next month, is one of the leading operating surgeons on the other side of the Border. Qualifying M.D., C.M., of Aberdeen in 1865, Sir Alexander Ogston has maintained a distinguished connection with that city ever since, and, having done much active work in connection with the Royal Infirmary, now holds the post of consulting surgeon there. He is also Emeritus Regius Professor of Surgery in the University and Surgeon-in-Ordinary to H.M. the King in Scotland; the latter post was also held by him in connection with the Courts of the late King Edward VII. and Queen Victoria.

High-frequency treatment is a method which came in with a great "boom," and comparatively quickly lost the confidence of the medical profession in spite of the fact that in certain disorders it can be used with advantage. Several workers have persisted in their allegiance to the method, amongst them Dr. Thomas D. Luke, M.D., F.R.C.S., who in a recent number of the *Practitioner* (June 1914) holds a brief for the treatment of various diseases by high-tension currents of electricity. Acknowledging the harm done to the high-frequency treatment by its initial boom, Dr. Luke, who for some years has been a recognised authority on physical remedies of various kinds, points out that with careful use both auto-condensation and the high-frequency effluve are extremely useful in selected cases of neurasthenia, insomnia, neuritis, and various disturbances of metabolism.

The vacant physicianship at Guy's Hospital has been filled by the promotion of Mr. A. P. Beddard, M.D., F.R.C.P., until now assistant physician; and in his place Mr. E. P. Poulton, M.D., M.R.C.P., has been elected to the junior appointment. Both these physicians are Oxford graduates, and the latter has lately published some important researches on diabetes.

These have been connected with the estimation of impurities, particularly carbonic dioxide gas, in the air of the lung cells in cases of this disease. Dr. Poulton's researches seem to show that the following up of this line of research may enable a definite standard to be constructed by which the alveolar air analysis will be indicative of the stage of the disease. Dr. Poulton did his clinical work at Guy's, where he won the Treasurer's gold medal in medicine; he was also awarded the Radcliffe travelling scholarship of Oxford University.

Dr. T. Claye Shaw, M.D., F.R.C.P., whose plea for the degenerate, made recently to the Harveian Society, has attracted a good deal of attention, is one of our veteran medico-psychologists, and of recent years has written on many subjects that concern the mental welfare of the race, in addition to his work on purely technical matters. Dr. Claye Shaw is well known to "Bart.'s" men, as he lectured at that institution on psychological medicine for many years, and now holds the post of Emeritus Lecturer on his special subject there. His idea appears to be that the dull people form a sort of make-weight to those of quicker brain.

The remarkable suggestion that mankind had at one time a blue skin was recently made in course of a discussion at the Royal Society of Medicine by Dr. E. A. Cockayne, one of the assistant physicians at the Middlesex Hospital. The debate in question concerned the meaning of the blue spots which sometimes occur on the skin, particularly in Mongolian races, and which are, therefore, commonly referred to as "Mongolian blue spots." From the evidence then brought forward it would appear that when blue spots were characteristic of man's appearance this extraordinary ornamentation appeared chiefly on the back, face, and dorsal surfaces of the limbs. Dr. E. A. Cockayne is also physician to out-patients at the Victoria Hospital for Children.

Dr. Gustave Monod, M.D. Paris, who has achieved some distinction by being the only French physician for several hundred years who has become a Member of the London Royal College of Physicians, is to be congratulated on his appointment as physician to the Thermal Hospital at Vichy. It is understood that this is the first instance in which a Member of the Royal College of Physicians has become head of a French hospital.

The numerous friends and acquaintances of Dr. Maccormac, the medical superintendent of St. James' Infirmary, Balham, will be grieved to hear that he is seriously ill with enteric fever. He has recently returned from his holidays, and probably contracted the fever during a long walking tour from the north of France to Lourdes, and thence on to the Pyrenees.

